# Glucocorticoid toxicity reduction with mepolizumab using the Glucocorticoid Toxicity Index

**DOI:** 10.1183/13993003.00160-2021

**Published:** 2022-01-20

**Authors:** P. Jane McDowell, John H. Stone, Yuqing Zhang, Kirsty Honeyford, Louise Dunn, R. Jayne Logan, Lorcan P.A. McGarvey, Claire A. Butler, Liam G. Heaney

**Affiliations:** 1 Wellcome-Wolfson Centre for Experimental Medicine, School of Medicine, Dentistry, and Biological Sciences, Queen's University Belfast, Belfast, UK; 2 Division of Rheumatology, Allergy, and Clinical Immunology, Massachusetts General Hospital, Harvard Medical School, Boston, MA, USA; 3 Dept of Respiratory Medicine, Belfast City Hospital, Belfast Health and Social Care Trust, Belfast, UK

## Abstract

**Background:**

Reduction in glucocorticoid exposure is the primary benefit of new biologic treatments in severe asthma, but there is currently no evidence that reduction in glucocorticoid exposure corresponds to a proportionate reduction in associated toxicity.

**Objectives:**

To use the validated Glucocorticoid Toxicity Index (GTI) to assess change in glucocorticoid toxicity after 12 months treatment with mepolizumab, and compare toxicity change to glucocorticoid reduction and change in patient-reported outcome measures (PROMs).

**Methods:**

A longitudinal, real-world prospective cohort of 101 consecutive patients with severe asthma commenced on mepolizumab in a specialist UK regional severe asthma clinic. GTI toxicity assessment, cumulative glucocorticoid exposure and PROMs were recorded on commencing mepolizumab (V1), and after 12 months treatment (V2).

**Results:**

There was significant reduction in oral glucocorticoid exposure (V1 median 4280 mg prednisolone per year (interquartile range 3083–5475 mg) *versus* V2 2450 mg prednisolone per year (1243–3360 mg), p<0.001). Substantial improvements in individual toxicities were observed, but did not correlate with oral glucocorticoid reduction. Mean±sd GTI aggregate improvement score (AIS) was −35.7±57.8 with a wide range in toxicity change at individual patient level (AIS range −165 to +130); 70% (71 out of 101) had a reduction in toxicity (AIS <0); 3% (three out of 101) had no change (AIS=0); and 27% (27 out of 101) an increase in overall toxicity. 62% (62 out of 101) of patients met the AIS minimally clinically important difference of ≤−10, but AIS did not correlate with glucocorticoid reduction or change in PROMs.

**Conclusion:**

Mepolizumab resulted in substantial oral glucocorticoid reduction, but this did not correlate with reduction in oral glucocorticoid toxicity, which varies widely at the individual patient level. Oral glucocorticoid reduction is not a comprehensive measure of response to mepolizumab.

## Abstract 2

**Anti-T2 biologics decrease glucocorticoid requirements in severe eosinophilic asthma, but glucocorticoid reduction does not correlate with glucocorticoid toxicity reduction in individuals. Assessment of glucocorticoid toxicity reduction is crucial when considering response to biologics.** https://bit.ly/3goyIRd

## Introduction

Biological therapies targeting type-2 (T2) inflammatory pathways in severe eosinophilic asthma (SEA) are effective in facilitating a decrease in systemic glucocorticoid exposure by reducing asthma exacerbations by ∼50%, [1–4] and facilitating maintenance oral glucocorticoid weaning [5–7].

A key anticipated benefit of biologics is glucocorticoid toxicity reduction, given the frequently occurring, multisystem adverse events known to have an increased incidence in individuals with severe asthma exposed to glucocorticoids, when compared to matched mild–moderate asthmatics and nonasthmatic controls [8–11]. Reduction in glucocorticoid exposure is the pragmatic primary outcome of clinical trials for biologics in SEA, but there is currently no evidence that reduction in glucocorticoid exposure produces a corresponding reduction in toxicity.

Mepolizumab, an anti-interleukin-5 monoclonal antibody, is used in the treatment of SEA to inhibit the recruitment, activation and longevity of eosinophils in the airways [1, 5, 12]. In the UK, access to mepolizumab and other biologics in SEA is governed by the National Institute for Health and Care Excellence (NICE), which advises that the decision of continuing or discontinuing biological therapy is based on the determination of an “adequate response” defined as a “clinically significant reduction in glucocorticoid-requiring exacerbations” (50% reduction for mepolizumab) or a “clinically significant reduction in continuous oral glucocorticoids” [13–16]. There is no clear guidance on what constitutes a “clinically significant reduction”, but accepting that the major problem with systemic glucocorticoid are the well-recognised side-effects, toxicity reduction is a central issue which is becoming more widely acknowledged [17].

Using the Glucocorticoid Toxicity Index (GTI) [18], we have shown previously that quantification of pre-biologic glucocorticoid toxicity in SEA patients with substantial systemic glucocorticoid exposure demonstrates wide variation at the individual patient level [19]. Here, we use the GTI to quantify change in glucocorticoid-associated toxicity in a SEA patient population treated with mepolizumab over a 12-month period in the course of routine clinical care. We evaluate the relationships between glucocorticoid toxicity change, variation in cumulative glucocorticoid dose and asthma outcome measures typically used to define a treatment response to biological therapies.

## Methods

### Design

This was a prospective, single-centre, observational cohort of glucocorticoid exposure and glucocorticoid toxicity change in sequential mepolizumab-treated SEA patients in a regional severe asthma specialist clinic in the UK. The GTI enabled systematic assessment of glucocorticoid toxicity using medical history, medication review, physical examination and routine blood tests. Patients underwent GTI assessment on commencing mepolizumab (V1), and after 12 months treatment (V2). Baseline pre-biologic glucocorticoid-associated toxicity burden has previously been described in this cohort with a mean±sd toxicity of 177.5±73.7 points [19].

Asthma-specific and quality-of-life patient-reported outcome measures (PROMs) were completed at V1 and V2, including the Mini Asthma Quality of Life Questionnaire (mini-AQLQ), St George's Respiratory Questionnaire (SGRQ), Asthma Control Questionnaire 5 (ACQ5), Hospital Anxiety and Depression score (HADS) and EuroQol-5D5L (see supplementary material for further information). Asthma nurse specialists evaluated patients every 4 weeks to administer mepolizumab, review medication use and asthma control (symptoms, lung function and exacerbation history). After 3 months of mepolizumab, glucocorticoid dose was reduced in those receiving maintenance glucocorticoids in accordance with clinical protocol (supplementary material) [20, 21].

### Patients

All patients had SEA as defined by the Global Initiative for Asthma guidelines [22] and were eligible to receive mepolizumab (100 mg subcutaneously every 4 weeks) according to NICE guidelines [16] (supplementary material). As part of the eligibility criteria, all underwent adherence evaluations to standard-of-care inhaled treatment using digital monitoring technology [23], medicines possession ratio and number of rescue glucocorticoid courses required. These data were retrieved through electronic healthcare prescribing records [24].

### GTI scoring

The GTI [18] and its online application (GTI 2.0 app© 2016, 2018, Massachusetts General Hospital, all rights reserved [19]) were developed as a practical tool to systematically measure glucocorticoid toxicity and assess change over time. The GTI captures the full sweep of glucocorticoid toxicity and permits the app to apply systematically determined relative weights to each toxicity item. Development and validation of the GTI, including its minimal clinically important difference (MCID), have been reported previously [18, 19]. In brief, the cumulative worsening score (CWS) is an additive record of glucocorticoid-related toxicities experienced by a patient from baseline. The score is always ≥0. A CWS of 0 indicates that no new glucocorticoid toxicities were present at V2. The higher the CWS, the greater the number of new toxicities encountered. The maximum possible CWS is 439. The aggregate improvement score (AIS) records present toxicity, allowing both improvement and worsening. A positive score indicates increased total toxicity, whereas a negative score reflects reduced toxicity. The MCID for the AIS is −10 [19]. The minimum AIS possible is −346, the maximum +439.

### Statistical analysis

Numbers and frequencies were reported for categorical variables. Continuous variables were reported as mean±sd or median (interquartile range (IQR)), as appropriate, considering their distributions. We calculated the differences between V2 and V1. The distributions of these differences are normal, therefore we used paired t-tests to obtain the mean of the differences and their 95% confidence intervals. We used a logistic regression model to examine the association between measures of glucocorticoid use and the AIS MCID. In addition, we assessed changes of each patient's reported outcome in relation to the AIS MCID using the logistic regression model. This model was adjusted for age and sex. We provide the odds ratio and 95% confidence interval for each point estimate. We created a scatterplot of AIS against percentage reduction in glucocorticoid exposure and calculated the Spearman correlation coefficient. For the data reported in table 4, we divided the CWS into two categories, namely CWS=0 and CWS>0. These categories signify, on one hand, no worsening of glucocorticoid toxicity between V1 and V2, and on the other, the development of new toxicity between these time points. We then examined the relationships of measures of glucocorticoid use as well as the patients reported outcomes to CWS worsening using logistic regression, adjusted for age and sex. Statistical analysis was undertaken on SPSS version 25 (IBM, Chicago, IL, USA).

## Results

**FIGURE 1:**
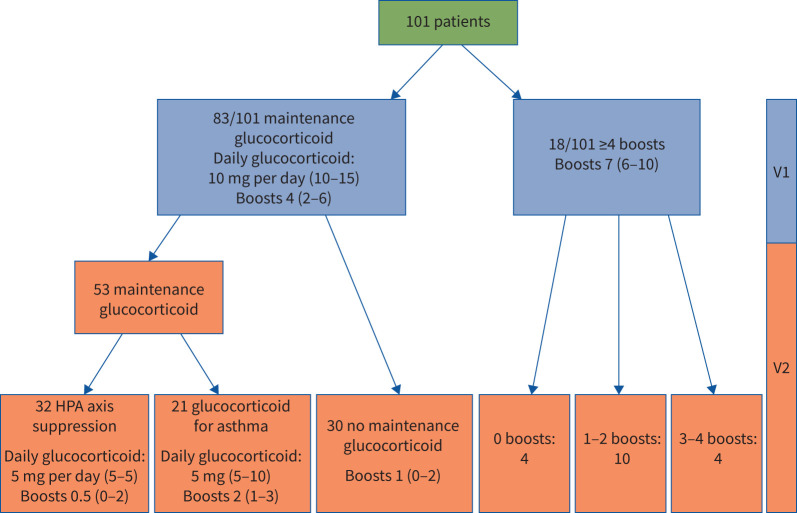
Clinical outcome of cohort and oral glucocorticoid exposure (n=101) from baseline (V1) and after 12 months of mepolizumab (V2). HPA: hypothalamic–pituitary–adrenal.

Between April 2017 and November 2019, GTI assessment was undertaken on sequential patients commencing mepolizumab (n=101; table 1, supplementary table E1) and after 12 months treatment. Clinical disposition after 12 months of mepolizumab is shown in figure 1. Of the 83 participants on maintenance prednisolone, 30 withdrew prednisolone completely and 32 had hypothalamic–pituitary–adrenal (HPA) axis suppression precluding complete withdrawal, with only 21 requiring maintenance prednisolone for asthma control.

**TABLE 1:** Clinical and demographic features of patients with severe eosinophilic asthma on commencing mepolizumab (V1)

**Patients**	101
**Age (years)**	54.4±11.9
**Female**	58 (57.4)
**Smoking status**	
Never-smoker	60 (61.9)
Ex-smoker	35 (36.1)
Current smoker	2 (2.1)
**BMI (kg·m^−2^)**	30.5±5.8
**Age at onset of asthma (years)**	30 (14–40)
**Atopic disease**	47 (48.5)
**Glucocorticoid rescue courses per 12 months**	5 (2–7)
**Cumulative prednisolone dose (mg) per 12 months**	4280 (3085–5475)
**ED visits per 12 months**	120/39 patients
**Hospital admissions per 12 months**	60/29 patients
**Ever been ventilated**	14 (13.9)
**FEV_1_ % predicted** **^#^**	68.9±19.0
**FEV_1_/FVC %**	61.9±14.6
***F*_eNO_ (ppb)**	35 (20.3–56.8)
**Blood eosinophil (cells·µL^−1^)**	280 (100–600)
**Highest blood eosinophils (cells·µL^−1^) in medical record**	860 (600–1300)
**IgE (kU·L^−1^)**	117.5 (42.3–351.3)
**Bone density T score (hip)**	−0.40±1.1
**Bone density T score (spine)**	−0.72±1.1
**Daily prednisolone**	83 (82.2)
**Daily prednisolone dose (mg) (n=83)**	10 (10–15)
**ICS daily dose (BDP µg equivalent)**	2000 (2000–2000)
**Long-acting β-agonist**	101 (100)
**Long-acting muscarinic antagonist**	35 (36.5)
**Leukotriene receptor antagonists**	51 (51)
**Theophylline**	38 (38.4)
**Nebulised bronchodilators**	33 (34.4)
**Maintenance macrolide**	7 (7)
**ACQ5**	2.6±1.3
**Mini-AQLQ**	3.6±1.4
**SGRQ**	55.8±20.9
**EuroQoL-5D5L index**	0.63 (0.4–0.8)
**EuroQoL-5D5L health scale**	65 (50–75)
**HADS depression**	6 (3–11)
**HADS anxiety**	8 (5–14)

Data are presented as n, mean±sd, n (%) and median (interquartile range). Further descriptive statistics for these variables can be found in supplementary table E1. BMI: body mass index; ED: emergency department; FEV_1_: forced expiratory volume in 1 s; FVC: forced vital capacity; *F*_eNO_: exhaled nitric oxide fraction; ICS: inhaled corticosteroid; BDP: beclomethasone dipropionate; ACQ: Asthma Control Questionnaire; AQLQ: Asthma Quality of Life Questionnaire; SGRQ: St George's Respiratory Questionnaire; HADS: Hospital Anxiety and Depression Scale. ^#^: using Global Lung Initiative 2012 predictive values.

The cohort's baseline systemic glucocorticoid exposure was substantial, consistent with NICE access requirements for biologic therapy eligibility [16]. 18 out of 101 participants had four or more glucocorticoid rescue courses in the 12 months preceding mepolizumab therapy, with the remainder on maintenance prednisolone (figure 1). In the year before V1, median (IQR) glucocorticoid exposure was 4280 (3082.5–5475.0) mg prednisolone per year (median (IQR) daily dose 11.7 (8.4–15.0) mg), reducing to 2450 (1242.5–3360) mg prednisolone per year (daily dose 6.7 (3.4–9.2) mg) on mepolizumab at V2 (p<0.001), representing a median (IQR) 44.5% (34–64%) reduction in cumulative glucocorticoid exposure. Comparing the 12 months prior to commencing mepolizumab (V1) with the 12 months on mepolizumab treatment (V2), asthma exacerbations declined (median (IQR) 5 (2–7) *versus* 1 (0–2), p<0.001) with an 88% reduction in emergency department visits and 72% reduction in hospital admissions. There was no difference in pre-bronchodilator spirometry or exhaled nitric oxide fraction between V1 and V2 (table 2, supplementary table E2).

**TABLE 2:** Clinical and patient reported outcomes at baseline (V1) and after 12 months mepolizumab treatment (V2)

	**V1**	**V2**	**Toxicity change at V2 (95% CI)**	**p-value**
**Total ED attendances past 12 months**	120/39 patients	15/10 patients	−1.05 (−1.55–−0.55)	<0.001
**Hospital admissions past 12 months**	60/29 patients	17/11 patients	−0.42 (−0.71–−0.14)	0.004
**FEV_1_ % predicted**	68.9±19.0	70.1±21.7	1.16 (−1.69–4.01)	0.42
**FVC % predicted**	85.9±16.0	86.5±17.4	0.79 (−1.66–3.25)	0.52
**FEV_1_/FVC**	61.9±14.6	62.1±16.9	0.23 (−2.51–2.97)	0.87
***F*_eNO_ (ppb)**	35 (20–57)	38 (24–68)	0.01 (−9.56–9.58)	0.99
**Blood eosinophils (cells·µL^−1^)**	280 (100–600)	60 (40–100)	−280 (−340–−210)	<0.001
**BMI (kg·m^−2^)**	30.5±5.8	29.9±5.5	−0.66 (−0.27–−1.06)	0.002
**BP systolic (mmHg)**	130.3±16.1	129.3±18.0	−0.96 (−3.58–1.66)	0.003
**LDL (mmol·L^−1^)**	2.8±0.9	2.6±0.9	−0.14 (−0.25–−0.02)	0.02
**Total cholesterol (mmol·L^−1^)**	5.3±1.1	5.0±1.1	−0.31 (−0.45–−0.17)	0.006
**HbA1c (mmol·mol^−1^)**	41 (37–46)	39 (35–43)	−2.68 (−3.75–−1.62)	<0.001
**Mini AQLQ overall^#^**	3.6±1.4	4.5±1.6	0.88 (0.61–1.16)	<0.01
**SGRQ overall^¶^**	55.8±20.9	43.4±23.2	−12.81 (−16.50–−9.12)	<0.01
**ACQ5 total^#^**	2.6±1.3	1.7±1.2	−0.89 (−1.15–−0.63)	<0.01
**HADS anxiety^+^**	8 (5–13.5)	7 (4–11)	−1.02 (−1.68–−0.36)	<0.01
**HADS depression^+^**	6 (3–11)	5 (2–10)	−1.04 (−1.76–−0.32)	<0.01
**EuroQoL-5L5D index**	0.63 (0.4–0.8)	0.71 (0.4–0.9)	0.05 (0.003–0.10)	0.04
**EuroQoL-5L5D health scale**	65 (50–75)	75 (56–85)	9.68 (5.87–13.50)	<0.01

Data are presented as n, mean±sd and median (interquartile range), unless otherwise stated. ED: emergency department; FEV_1_: forced expiratory volume in 1 s; FVC: forced vital capacity; *F*_eNO_: exhaled nitric oxide fraction; BMI: body mass index; BP: blood pressure; LDL: low-density lipoprotein; HbA1c: glycated haemoglobin; AQLQ: Asthma Quality of Life Questionnaire; SGRQ: St George's Respiratory Questionnaire; ACQ: Asthma Control Questionnaire; HADS: Hospital Anxiety and Depression Scale. **^#^**: minimal clinically important difference (MCID) 0.5; **^¶^**: MCID 4 units; **^+^**: 0–7 (normal), 8–10 (mildly disturbed), >11 (psychiatric impairment). Further descriptive statistics for these variables can be found in supplementary table E2.

### Toxicity change

Change in specific toxicities are displayed in table 2 and supplementary table E2. Mean body mass index (BMI) declined between V1 and V2, with 38% (38 out of 101) of patients having a reduced BMI, 5% (five out of 101) an unchanged BMI and 57% (58 out of 101) an increased BMI at V2. The correlation between BMI change and percentage reduction in prednisolone exposure was not significant (ρ=0.14, p=0.18).

#### Glycaemic control

On commencing mepolizumab, 13 patients were prescribed antidiabetic medication with a significant fall in glycated haemoglobin (HbA1c) seen at V2 (supplementary figure E1). Of the 88 out of 101 patients not known to have diabetes at V1, 64.8% (57 out of 88) had an HbA1c in the normal range, 27.3% (24 out of 88) had an HbA1c in the high-risk pre-diabetic range and 8% (seven out of 88) had an HbA1c in the diabetic range. There was no significant correlation between change in HbA1c and percentage reduction in cumulative glucocorticoid exposure.

#### Lipids

27 (26.7%) of the cohort received antilipid medication at V1 with median (IQR) low-density lipoprotein (LDL) concentrations of 2.8 (1.6–3.8) mmol·L^−1^ and 2.4 (1.5–3.2) mmol·L^−1^ at V2, (p=0.07); 24 out of 27 patients had no change in antilipid medication, two out of 27 had a decrease and one out of 27 had an increase in antilipid medication from V1 to V2. There was no correlation between LDL and percentage change in cumulative glucocorticoid exposure from V1 to V2.

#### Infection

In the 12 months prior to starting mepolizumab, 18.6% (18 out of 97) of patients had candidiasis (oral or vaginal) or herpes zoster without neuralgia or ophthalmic involvement, declining to 5% (five out of 101) at V2 (p=0.002). Herpes zoster with neuralgia or eye involvement was described in two patients in the 12 months before V1, but no cases were identified between V1 and V2. In the 12 months prior to initiation of mepolizumab therapy, 18.8% (19 out of 101) patients had at least one hospitalisation for infection. This percentage declined to 7.9% (eight out of 101) at V2 (p=0.04).

#### Skin

A significant reduction in skin toxicity was apparent at V2. Acneiform rash was reported by six out of 101 patients at V1 and two out of 101 at V2 (p=0.046); easy bruising was reported by 68 out of 101 patients at V1 and 54 out of 101 at V2 (p=0.003); and striae were reported by 35 out of 101 at V1 and 20 out of 101 at V2 (p=0.003). Of the 58 female patients in this cohort, 25 (43.1%) had hirsutism at V1, but only nine (15.5%) at V2 (p≤0.001).

#### Neuropsychiatric disturbance

The most common toxicity at mepolizumab initiation was neuropsychiatric disturbance*.* Depressive symptoms were highly prevalent at baseline and continued to be an important issue through to V2 (supplementary table E3). At V2, 45 (44.6%) out of 101 patients reported fewer depressive symptoms and only five (5%) out of 101 reported increased symptoms.

Mood disturbance of irritability or mood elevation improved at V2, with 43 (42.6%) out of 101 patients reporting mood improvement and only eight (7.9%) out of 101 reporting worsening mood disturbance. Two patients had experienced previous psychotic episodes while receiving high-dose glucocorticoids at V1; there were no episodes described at V2.

The majority of patients (56 (55%) out of 101) reported some degree of insomnia at initiation of mepolizumab. At V2, 30.7% (31 out of 101) had improved insomnia and 8.9% (nine out of 101) had worsened insomnia symptom (see supplementary table E4 for further detail on neuropsychiatric change).

### Patient-reported outcomes

Changes in PROMs are shown in table 2 and supplementary table E5. For ACQ5, 59% (58 out of 98 paired questionnaires) of patients met or exceeded the ACQ5 MCID (≥0.5), and 60% (58 out of 96) and 71% (68 out of 96) met the MCID for the mini-AQLQ and SGRQ, respectively. At V2, patients reported improvement in their overall health status measured with the EuroQoL-5L5D health scale.

Overall anxiety and depression scores improved at V2 (table 2); 10% fewer patients were in the severe HADS category for anxiety (score ≥11) and 9% more were in the normal range. There was an 11% decrease in the number of patients in the HADS severe depression category (score ≥11), a 4% increase in the moderate category and an 8% increase in the number of patients in the normal range. The GTI depression score correlated well with the HADs depression scores at V1 (ρ=0.6, p<0.001) and V2 (ρ=0.53, p<0.001) (supplementary table E6).

There was a weak correlation between percentage prednisolone reduction and change in mini-AQLQ (ρ=−0.25, p=0.015); however, there was no correlation with change in ACQ5, SGRQ, HADS depression or HADS anxiety scores.

### Quantifying change in toxicity over time

**FIGURE 2:**
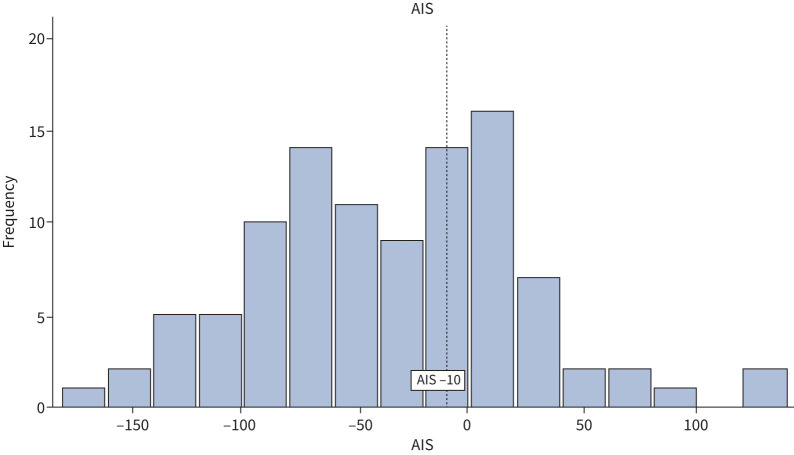
Distribution of aggregate improvement score across the cohort (n=101): 70% (71 out of 101) of patients had an aggregate improvement score (AIS) <0, 3% (three out of 101) an AIS=0 and 27% (27 out of 101) an AIS >0.

The mean±sd AIS for this cohort was −35.7±57.8. The degree of toxicity change varied widely at the individual patient level (AIS range −165 to +130; figure 2). A reduction in toxicity (AIS<0) was seen in 70% (71 out of 101) of the cohort, while 3% (three out of 101) had no change (AIS=0) and 27% (27 out of 101) had an increase (AIS≥1). AIS did not correlate with baseline glucocorticoid-associated toxicity score or change in PROMs (supplementary figure E2). Toxicity change (AIS) did not have a significant linear correlation with oral glucocorticoid reduction from V1 to V2 when measured as percentage reduction in glucocorticoid, cumulative reduction in glucocorticoid (mg) or reduction in glucocorticoid rescue courses (supplementary figure E3).

Of the 101 patients, 62 patients met the AIS MCID of ≤−10 (mean±sd AIS −71.4±37.2, range −165 to −10; figure 2) and 39 patients did not (AIS 21.1±34.3, −9 to +130). Participants who met the MCID had fewer glucocorticoid rescue courses for exacerbations at V2 (one rescue course (IQR 0–2) *versus* two rescue courses (0–3); OR 0.73, 95% CI 0.55–0.98; p=0.04) and a greater percentage reduction in prednisolone exposure from V1 to V2 (48.6%, 34.8–70.0% *versus* 40.8%, 26.1–57.2%; OR 0.80, 95% CI 0.66–0.96; p=0.02). However, the proportion of patients receiving maintenance glucocorticoid and daily dose did not differ in those who met the MCID and those who did not (table 3). Baseline glucocorticoid-associated toxicity on commencing mepolizumab and change in PROMs did not differ in those who met the AIS MCID and those who did not (table 3).

**TABLE 3:** Associations of oral glucocorticoid use and patient reported outcomes with achieving aggregate improvement score (AIS) minimal clinically important difference (MCID) (≤−10)

	**AIS ≤−10**	**AIS >−10**	**Change across AIS MCID** **Crude OR (95% CI)**	**Change across AIS MCID** **Age- and sex-adjusted OR (95% CI)**
**Patients**	62	39		
**Glucocorticoid use across AIS MCID**				
Median daily prednisolone (mg) V1	11.2 (8.4–15.0)	12.3 (8.7–14.6)	0.99 (0.94–1.04)	0.99 (0.94–1.04)
Median daily prednisolone (mg) V2	6.5 (2.2–8.5)	7.8 (4.0–9.4)	0.96 (0.91–1.03)	0.96 (0.91–1.03)
Receiving maintenance prednisolone V1	49 (79.0)	34 (87.2)	0.55 (0.18–1.70)	0.58 (0.18–1.80)
Receiving maintenance prednisolone V2	27 (43.5)	25 (64.1)	0.45 (0.20–1.02)	0.40 (0.17–0.96)
Number of glucocorticoid rescue courses V1	5 (2–7)	5 (3–8)	0.96 (0.85–1.08)	0.96 (0.85–1.08)
Number of glucocorticoid rescue courses V2	1 (0–2)	2 (0–3)	0.73 (0.55–0.98)	0.70 (0.52–0.94)
Decrease annual prednisolone (mg)	−1925 (−1400–−2888)	−1727.5 (−2527–−1200)	0.98 (0.94–1.02)	0.98 (0.94–1.01)
Percentage decrease annual prednisolone	48.6 (34.8–70.0)	40.8 (26.1–57.2)	0.80 (0.66–0.96)	0.80 (0.66–0.97)
Baseline glucocorticoid-associated toxicity score	165.5 (124–211)	164 (129–229)	1.00 (0.99–1.01)	1.00 (0.99–1.01)
**Change in PROMs across AIS MCID**				
Mini-AQLQ	0.82 (−0.7–1.6)	0.94 (0.03–−1.7)	0.91(0.67–1.23)	0.93 (0.68–1.28)
SGRQ	−13.3 (−23.4–−4.6)	−9.9 (−15.8–4.6)	0.99 (0.97–1.01)	0.99 (0.96–1.01)
ACQ5	−0.6 (−1.4–0.0)	−0.8 (−2.2–0.0)	1.26 (0.91–1.75)	1.29 (0.92–1.81)
HADS anxiety	−1.0 (−3.0–0.8)	−1.0 (−2.0–1.5)	0.94 (0.83–1.07)	0.95 (0.83–1.08)
HADS depression	−1.0 (−3.0–1.0)	−1 (−2.0–0.3)	0.95 (0.85–1.07)	0.94 (0.84–1.06)
EuroQoL-5L5D index	0.0 (−0.08–0.1)	0.02 (−0.06–0.1)	1.29 (0.24–6.86)	1.43 (0.26–7.99)
EuroQoL-5L5D health scale	5 (−1–16.3)	12 (−4–24)	1.00 (0.97–1.02)	1.00 (0.97–1.02)

Data are presented as n, median (interquartile range) or n (%), unless otherwise stated. V1: at commencement of mepolizumab; V2: after 12 months treatment; PROMs: patient-reported outcome measures; AQLQ: Asthma Quality of Life Questionnaire; SGRQ: St George's Respiratory Questionnaire; ACQ: Asthma Control Questionnaire; HADS: Hospital Anxiety and Depression Scale.

**TABLE 4:** Associations of oral corticosteroid use and patient reported outcome measures (PROMs) with the risk of developing new glucocorticoid-toxicities (cumulative worsening score (CWS) ≥1)

	**CWS 0**	**CWS ≥1**	**Change across CWS 0 and ≥1** **Crude OR (95% CI)**	**Change across CWS 0 and ≥1** **Age- and sex-adjusted OR (95% CI)**
**Patients**	38	63		
**Glucocorticoid use across CWS 0 and CWS ≥1**				
Daily prednisolone (mg) V1	11.2 (9.0–16.7)	11.7 (8.1–14.6)	0.99 (0.95–1.04)	1.00 (0.95–1.05)
Daily prednisolone (mg) V2	6.71 (3.83–9.88)	6.71(3.07–9.06)	1.00 (0.94–1.06)	1.00 (0.94–1.06)
Receiving maintenance prednisolone V1	32 (84.2)	51 (81.0)	0.80 (0.27–2.34)	0.77 (0.26–2.29)
Receiving maintenance prednisolone V2	20 (52.6)	32 (50.8)	0.45 (0.20–1.02)	0.40 (0.17–0.96)
Number of glucocorticoid rescue courses V1	5.0 (2.0–6.0)	5.0 (2.5–7.0)	1.04 (0.92–1.18)	1.05 (0.93–1.19)
Number of glucocorticoid rescue courses V2	1.0 (0.0–2.0)	2.0 (0–2)	1.42 (1.03–1.95)	1.44 (1.04–2.01)
Decrease annual prednisolone (mg) (V2–V1)	−1881 (−2806–−1374)	−1760 (−2723–−1393)	1.01 (0.98–1.05)	1.01 (0.97–1.05)
Percentage decrease annual prednisolone	−42.0 (−62.9–−34.2)	−44.6 (−66.7–−32.2)	1.01 (0.85–1.20)	1.01 (0.85–1.29)
Baseline glucocorticoid-associated toxicity score	168 (124–190)	165 (129–235)	1.00 (1.00–1.01)	1.00 (1.00–1.01)
**Change in PROMs across CWS 0 and CWS ≥1**				
Mini-AQLQ	0.77 (0.1–1.5)	1.0 (−0.1–1.8)	1.01 (0.75–1.38)	1.03 (0.75–1.41)
SGRQ	−14.0 (−21.0–−5.3)	−11.2 (−20.9–2.9)	1.01 (0.99–1.03)	1.01 (0.98–1.03)
ACQ5	−0.6 (−1.8–−0.2)	−0.6 (−1.8–0.0)	1.04 (0.76–1.43)	1.02 (0.74–1.41)
HADS anxiety	−1.0 (−2.5–1.0)	−1.0 (−3.0–0.5)	0.97 (0.85–1.10)	0.96 (0.84–1.09)
HADS depression	−1.0 (−3.0–1.0)	−1.0 (−3.0–1.0)	1.03 (0.91–1.15)	1.02 (0.90–1.14)
EuroQoL-5L5D index	0.0 (−0.05–0.2)	0.0 (−0.07–0.1)	0.44 (0.08–2.32)	0.51 (0.09–2.77)
EuroQoL-5L5D health scale	7.5 (−5.0–20.0)	10 (0–20)	1.01 (0.98–1.03)	1.01 (0.99–1.04)

Data are presented as n, median (interquartile range) or n (%), unless otherwise stated. V1: at commencement of mepolizumab; V2: after 12 months treatment; AQLQ: Asthma Quality of Life Questionnaire; SGRQ: St George's Respiratory Questionnaire; ACQ: Asthma Control Questionnaire; HADS: Hospital Anxiety and Depression Scale.

**FIGURE 3:**
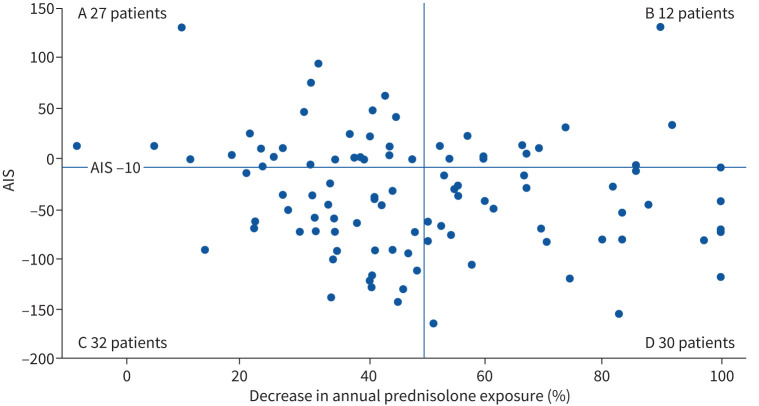
Aggregate improvement score (AIS) and reduction in annual prednisolone. Quadrant A: <50% reduction in prednisolone and AIS >−10; quadrant B: >50% reduction in prednisolone and AIS >−10; quadrant C: <50% reduction in prednisolone and AIS ≤−10; quadrant D: >50% reduction in prednisolone and AIS ≤−10. Spearman's correlation between AIS and percentage reduction in prednisolone is ρ=−0.18, p=0.08.

Defining a ≥50% reduction in annual glucocorticoid exposure from V1 to V2 as a clinically significant reduction, 32% (32 out of 101) of patients did not meet this response threshold, yet had a clinically meaningful reduction in glucocorticoid-associated toxicity (AIS ≤−10) (figure 3, quadrant C). In addition, 12% (12 out of 101) of patients who met this threshold of glucocorticoid reduction did not reach the MCID for glucocorticoid toxicity reduction (figure 3, quadrant B).

**FIGURE 4:**
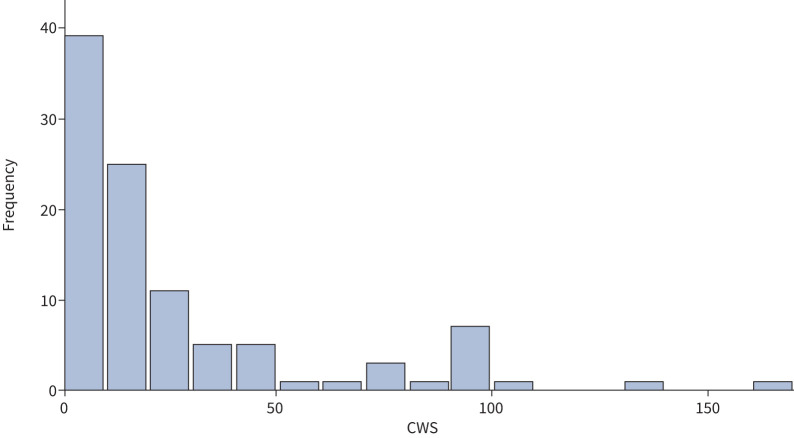
Distribution of cumulative worsening scores (CWS) across the cohort (n=101). The median CWS was 18, interquartile range 0–30, range 0–167.

Of the 101 patients in the cohort, 38% (38 out of 101) had a CWS of 0, indicating no new glucocorticoid toxicity at V2. The remaining 62% (63 out of 101) had a positive CWS, with individual scores ranging from 1 to 167 (figure 4). Neither baseline glucocorticoid-associated toxicity nor oral glucocorticoid reduction from V1 to V2 correlated with the occurrence of new toxicities as measured by the CWS (supplementary figure E4).

When comparing the 38 patients with no new toxicities (CWS 0) and the 63 patients with new toxicities (CWS ≥1), there was no difference in the proportion of patients on maintenance prednisolone at V1 or V2 and no difference in glucocorticoid reduction between the two groups (table 4). Although there was no difference in the number of glucocorticoid rescue courses for asthma exacerbations at V1, fewer exacerbations occurred in the group with no new toxicities at V2 (table 4). Both groups, CWS of zero and CWS ≥1, reported improvement in asthma symptoms and reported quality of life, with no difference between the groups.

Duration of asthma was associated with risk of CWS worsening (OR 1.03, 95% CI 1.00–1.06); however, no other demographic, anthropometric or baseline clinical factors were associated with the risk of CWS worsening. No associations were observed between these factors and AIS.

## Discussion

Previous studies have provided evidence for an increase in adverse events on exposure to oral glucocorticoids, and in asthma, have provided evidence for an increase in glucocorticoid-associated adverse events in oral glucocorticoid-exposed severe asthmatics when compared to matched controls (both mild–moderate asthmatics and nonasthmatic individuals) [8–11]. Here, we prospectively evaluated glucocorticoid reduction with 12 months mepolizumab in a real-world SEA cohort, and related this to changes in glucocorticoid toxicity and PROMs. The participants in the SEA cohort themselves acted as a control, with the comparison of baseline pre-biologics toxicity assessment, to toxicity assessment after 12 months treatment with a glucocorticoid-sparing medication. There was substantial reduction in glucocorticoid requirement, with the majority of patients having significant glucocorticoid-related toxicity reduction and PROM improvement; however, the relationship between glucocorticoid reduction and toxicity change was not linear.

Prior to initiation of mepolizumab, this cohort received substantial asthma treatment (table 1), yet continued to have evidence of T2 cytokine-driven disease. In keeping with clinical trials [1, 12], this real-world cohort had mepolizumab-enabled reduction of asthma exacerbations, hospitalisations, emergency department visits and a 44.5% reduction in 12-month cumulative glucocorticoid exposure.

While this considerable reduction in glucocorticoid led to a statistically significant reduction in BMI, systolic blood pressure, lipid profile and HbA1C, the overall changes were small and the clinical significance may seem questionable. However, it is recognised that elevation of these markers of vascular and metabolic dysfunction represents a cumulative continuum of risk [25–27]; therefore, even small reductions may be beneficial. In addition, glucocorticoid exposure is not the only influence on these measures, with calorie intake, exercise and daily activity reflecting complex behavioural and social habits emerging over years of having severe asthma and substantial glucocorticoid exposure. These factors may be more challenging to modify, and biologic-induced glucocorticoid reduction may need to be coupled with exercise programmes and dietary education to modify behaviour and facilitate reduction of metabolic risk. Medications to manage risk factors play a role, although importantly, GTI scoring takes medication changes into consideration when calculating toxicity change.

Improvement in mood disturbance was common, with double the number of patients reporting no depressive symptoms at V2, and a halving of those reporting severe depressive symptoms. Significant improvement in mood disturbance/mania and insomnia were also apparent. The aetiology of improved mood is multifactorial; reduction in glucocorticoid and improvements in disease control (ACQ5, SGRQ, mini-AQLQ) occur alongside improvement in overall health (EuroQoL-5D5L health scale) and reduction of total toxicity burden in the majority of patients.

Across the three respiratory PROMs, 60–70% of patients had improvement in symptoms that met the PROM MCID; there was a mean improvement in perceived overall health (EuroQoL-5D5L health scale). The improvement in PROMs correlated neither strongly nor significantly with reduction in glucocorticoid exposure or AIS toxicity change.

The wide distribution in AIS reflects varied toxicity change at the individual patient level after introduction of mepolizumab. Although the majority of patients had a significant reduction in toxicities, 30% had no change or worsening overall toxicity, which may not be surprising given that the median daily prednisolone dose at V2 was 6.7 mg·day^−1^ (IQR 3.4–9.2 mg·day^−1^), a dose at which glucocorticoid toxicity may continue to occur in some patients.

While these data demonstrate substantial reduction in glucocorticoid exposure, reduction of total toxicity burden and improvement in PROMS in the majority of mepolizumab-treated patients, the relationships between these three outcome measures are complex. Those who met the AIS MCID had fewer glucocorticoid rescue courses and a greater percentage glucocorticoid reduction at V2 than those who did not, but reduction in glucocorticoid dose did not correlate linearly with AIS. In fact, defining a 50% reduction in glucocorticoid exposure as an adequate glucocorticoid-sparing response to mepolizumab resulted in one-third of this cohort being defined as “nonresponders”, despite demonstration of a substantial improvement in glucocorticoid toxicity. Supplementary figure E5 further illustrates the point that not all patients will derive the same degree of toxicity reduction benefit from a given reduction in oral glucocorticoid treatment. Furthermore, a greater reduction in glucocorticoid did not equate to a reduced incidence of new glucocorticoid-toxicities when on mepolizumab.

Strengths of these data include the size of the severe asthma cohort, prospective data collection, and toxicity assessment using a standardised, validated instrument applied according to a specific protocol. Although components scored within the GTI were included as they are likely to exhibit change over the course of a reasonable period of clinical observation (*e.g.* between 3 months and 3 years), as is demonstrated in this manuscript with many glucocorticoid-associated toxicities improving, one potential limitation is the possibility that a time-lag from glucocorticoid reduction to toxicity improvement may exist with some toxicities, and in fact, once present, some toxicities may not be reversible. We hope to address this with further longitudinal assessment of toxicity change using the GTI, and anticipate that many of those patients with HPA-axis suppression will have adrenal recovery and be able to withdraw prednisolone completely.

In summary, observation of toxicity in this glucocorticoid-dependent cohort commencing a glucocorticoid-sparing therapy shows a significant reduction in glucocorticoid exposure, reduction in glucocorticoid-toxicity and improvement in PROMS in the majority of patients. Toxicity change varies widely at the individual patient level and glucocorticoid reduction is not a simple substitute for measuring toxicity change. As the purpose of glucocorticoid-sparing agents is to reduce toxicities resulting from the glucocorticoid, caution should be applied when solely using glucocorticoid reduction as a measure of response to mepolizumab.

## Supplementary material

10.1183/13993003.00160-2021.Supp1 **Please note:** supplementary material is not edited by the Editorial Office, and is uploaded as it has been supplied by the author.
Supplementary material ERJ-00160-2021.SUPPLEMENT

## Shareable PDF

10.1183/13993003.00160-2021.Shareable1 This one-page PDF can be shared freely online.
Shareable PDF ERJ-00160-2021.Shareable
