# Supplementary material for: Glucocorticoid toxicity reduction with mepolizumab using the Glucocorticoid Toxicity Index
Source: Eur Respir J. 2022 Jan 20;59(1):2100160. doi: 10.1183/13993003.00160-2021 (PMC8770919; doi:10.1183/13993003.00160-2021)
Supplement: Supplementary file 1 [file ERJ-00160-2021.SUPPLEMENT.pdf]

Glucocorticoid Toxicity Reduction with Mepolizumab using the Glucocorticoid Toxicity Index

P Jane McDowell, John H Stone, Yuqing Zhang, Kirsty Honeyford, Louise Dunn, Rebekah J Logan, Lorcan PA McGarvey, Claire A Butler, Liam G Heaney

Online Supplement

Supplementary statistical analysis:

Statistical analysis of the data was undertaken on SPSS version 25 (IBM, Chicago, IL). Categorical variables are summarized using counts and percentages. The association between baseline GC toxicity and AIS was examined using linear regression models, mean AIS for each quartile of baseline GC toxicity was compared both unadjusted and adjusted for age and sex. Pearson and Spearman's correlation coefficients were calculated for parametric and non-parametric data, respectively. The threshold for statistical significance throughout was a p value of less than 0.05.

Development of the GTI

The original version of the GTI (GTI 1.0) underwent preliminary validation by a multi-specialty group of nineteen physician experts(E1). These experts represented ten medical subspecialties: pulmonary medicine, rheumatology, paediatric rheumatology, nephrology, neurology, ophthalmology, dermatology, infectious disease, maternal-fetal medicine and psychiatry. The investigator group, which included 10 researchers from the United States and 9 from Europe, Canada, or Australia, employed group consensus methods and multi-criteria decision analysis(E2).

A full explanation of how the items required to give a complete quantification of GC-related toxicity were determined is outlined in reference E1. In brief, items to be included in the GTI scoring were determined on the following principles: 1) they occur commonly in GC-exposed patients (≥5% of such patients); 2) they are independent of other items in the GTI; 3) they are more likely to result from GC-exposure than from the underlying disease; 4) they are likely to change over a short period of time (i.e., to either improve or worsen over a period of at least three months); 5) they can be measured simply and non-invasively. A small number of the measured variables may be outside the usual clinical measurements for a given disease, but these are easy to collect and important for assessing GC toxicity as part of routine care. For example, HbA1C is not usually measured in severe asthma clinic, but is required to measure total toxicity in severe asthma patients treated with GC. This is a reasonable test to consider as standard care of a patient on chronic GC.

The GTI assigns relative weights that have been determined systematically to each toxicity item in the Composite Index. These weights were developed through multi-criteria decision analysis (MCDA)(E2) using pair-wise "forced-choice" methods within the 1000 Minds platform, all possible combinations of Composite Index items were ranked in order of toxicity severity. A point system reflecting the relative weight of each item in terms of toxicity was thereby derived.

Scoring the GTI

The fundamental principle with regard to the scoring approach is that an improvement in GC toxicity is given the same absolute weight as a worsening of GC toxicity of the same (but opposite) magnitude. GTI 2.0 uses the same positive weights derived in the initial GTI validation study and is the version now used in multi-center clinical trials. This approach generated two scores from the Composite Index data called: 1) the Aggregate Improvement Score (AIS); and, 2) the Cumulative Worsening Score (CWS). These scores and their rationales, outlined briefly below.

Aggregate Improvement Score (AIS) - In a disease such as GC-dependent asthma in which patients often have some baseline GC toxicity when starting a new treatment, the AIS is important in establishing that the new therapy is effective at diminishing any GC toxicity over time. With the AIS, toxicities are removed if they improve or resolve completely over longitudinal follow-up. Toxicities can also be added to the AIS over time if new or worsened GC toxicities occur. With the AIS, improvement in GC toxicity has the same absolute value as does worsening to the same degree in the opposite direction.

As an example, an increase in the body mass index (BMI) more than 5 BMI units to a BMI of greater than 25 is associated with an increase in the GTI score of +36 points. Conversely, a decrease in BMI of more than 5 BMI units towards a normal BMI is associated with an improvement in the score of -36 points. Thus, improvements in GC toxicity occurring over longitudinal follow-up are indicated by negative AIS values. If a new treatment agent facilitates reduced GC toxicity over time, the AIS will decrease over time.

Cumulative Worsening Score (CWS) - On the other hand, documentation of cumulative GC toxicity that occurs over the course of the trial or introduction of a new treatment is important, even if some toxicities are transient. The CWS is designed to assess the summative GC toxicity, regardless of whether the toxicity has lasting effects or resolves with time. New toxicities that occur are added to the CWS total, but toxicities that

resolve on follow-up are not removed. The CWS serves as a lasting record of any GC toxicity that has occurred, and can only increase or remain the same over time. Further information on the individual items scored in the GTI can be found in the supplement to reference E3.

Patient-Reported Outcome Measures in Severe Prednisolone-dependent Asthma

Patient-reported outcome measures (PROMs) in this study addressed change in quality-of-life and asthma severity.

Quality of life. The EuroQoL-5L5D Health scale is a visual analogue scale with which patients report their overall health,[E4] 100 is the best health possible and 0 reflects the worst overall health. The EuroQoL-5L5D Index value reflects impairment of activities of daily living, the closer the score is to 1, the less the impairment of daily living. The change in Index Value following an intervention allows evaluation of the health economics of the intervention through calculation of quality-adjusted life years.

The impact of asthma on quality of life was measured by mini-Asthma Quality of Life Questionnaire (mini-AQLQ) and St. George's Respiratory Questionnaire (SGRQ).[E5][E6] The mini-AQLQ consists of 15 questions scored on a scale of between 0 and 7, a lower score reflects greater impairment with MCID being met when the mini-AQLQ improves by 0.5 or more. The SGRQ assesses the impact of disease over the preceding 12 months, the higher the score the greater impact disease has on quality of life. The MCID is a score reduction of 4 units.

Asthma control. Asthma control was assessed by the five item asthma control questionnaire.[E7] An ACQ5 score of 0.75 reflects adequate asthma control, whereas a score of 1.5 or over signals inadequate asthma control. The MCID is a reduction in ACQ5 score of 0.5 or greater.[E8]

Anxiety and Depression. The Hospital Anxiety and Depression score (HADs) is a validated tool with outputs that quantify anxiety and depression. A score of 0-7 is defined as normal, 8-10 indicates mild anxiety and depression, 11 or more suggests requirement for psychiatric assessment.[E9][E10]

Oral GC weaning on commencing mepolizumab

After 12 weeks on mepolizumab, participants receiving maintenance oral glucocorticoids (GC) for asthma control began a process of GC weaning in a step-wise manner. Weaning was based on clinical response (symptoms and exacerbation history) until a dose of 5mg prednisolone/day, at which time hypothalamic-pituitary-adrenal (HPA) axis function was checked using 9am cortisol and short Synacthen testing given the high prevalence of HPA axis suppression in those treated with oral GCs. Further weaning was based on adequate HPA axis function, until complete GC withdrawal.

Table E1. Additional descriptive statistics for the clinical and demographic features of patients with severe eosinophilic asthma on commencing mepolizumab (V1).

	n	Minimum	Maximum	Mean	Std. Deviation	Median	Percentiles	
							25	75
Age (years)	101	18.0	76.0	54.4	11.9	55.0	47.0	63.0
BMI (kg/m²)	101	18.4	48.1	30.5	5.8	30.1	26.4	35.0
Age at onset of asthma (years)	95	2	70	28.7	17.4	30	14	40
GC rescue courses / 12 months	101	0	14	5.0	3.3	5	2	7
Cumulative prednisolone dose (mg) / 12 months	101	1400	22460	4773.4	3043.2	4280	3085	5475
ED visits / 12 months	100	0	14	1.2	2.5	0	0	1
Hospital admissions / 12 months	100	0	10	0.6	1.4	0	0	1
FEV1 % Predicted	100	36.3	110.7	68.9	19.0	68.1	54.2	84.6
FVC % Predicted	98	48.4	120.1	85.9	16.0	84.7	74.8	96.9
FENO (ppb)	100	16	281	49.1	47.7	35	20.3	56.8
Blood Eosinophil (cells/µL)	101	0	1850	360.0	320	280	100	600
Highest Blood Eosinophils (cells/µL) in medical record	101	0	16660	1190	1680	860	600	1300
IgE (kU/L)	100	2.0	10899.0	416.5	1174.1	117.5	42.3	351.3
Bone density T score (Hip)	94	-3.00	3.30	-0.40	1.1	-0.30	-1.13	0.30
Bone density T score (Spine)	94	-3.70	2.70	-0.72	1.1	-0.80	-1.43	0.03
Mean Daily prednisolone dose/12 month period (mg)	101	3.8	61.5	13.1	8.3	11.7	8.4	15.0
ICS Daily dose (BDP µg equivalent)	100	800	4000	1883.6	462.3	2000	2000	2000
ACQ5	101	0.0	5.8	2.6	1.3	2.6	1.8	3.5
Mini-AQLQ	100	1.0	6.7	3.6	1.4	3.6	2.5	4.8
SGRQ	99	7.2	94.7	55.8	20.9	57.5	40.0	70.4
Euro-QoL 5D5L Index	99	-0.47	1.00	0.60	0.31	0.63	0.41	0.8
Euro-QoL 5D5L Health Scale	96	15	100	62.4	19.3	65	50	75
HADS Depression	101	0	19	6.9	4.7	6	3	11
HADS Anxiety	101	0	20	8.8	5.3	8	5	14

Table E2. Additional descriptive statistics for the difference in clinical and patient reported outcomes from baseline (V1) to 12 months mepolizumab treatment (V2).

	N Valid	Minimum	Maximum	Mean	Std. Deviation	Median	Percentiles	
							25	75
Total ED attendances last 12 months in number of patients	100	-14	3	-1.05	2.52	0	-1	0
Hospital admissions last 12 months	99	-10	2	-0.42	1.42	0	0	0
FEV1 % Predicted	100	-33.3	46.0	1.16	14.37	1.6	-6.5	9.3
FVC % Predicted	96	-25.1	41.1	0.79	12.11	1.0	-6.6	7.4
FENO (ppb)	99	-137	273	0.01	46.37	3	-12	18
Blood Eosinophils cells/µL	101	-1790	550	-280.00	320.00	-210	-490	-40
BMI	100	-8.2	4.4	-0.66	1.99	-0.6	-1.8	0.7
BP Systolic (mmHg)	100	-53	37	-0.96	13.21	-2	-9	8
LDL (mmol/L)	100	-1.9	1.6	-0.14	0.58	-0.1	-0.4	0.2
Total Cholesterol (mmol/L)	100	-2.6	1.4	-0.31	0.70	-0.2	-0.7	0.1
HbA1c (mmol/mol)	101	-33	6	-2.68	5.38	-2	-5	1
Mini AQLQ Overall	96	-2.4	4.8	0.88	1.35	0.9	0.0	1.6
SGRQ Overall	96	-68.2	25.7	-12.81	18.21	-12.0	-21.0	-0.4
ACQ 5total	98	-5.0	4.0	-0.89	1.29	-0.6	-1.8	0.0
HADS: Anxiety	97	-10	8	-1.02	3.27	-1	-3	1
HADS: Depression	98	-11	9	-1.04	3.59	-1	-3	1
ED5L5D Index value	96	-0.57	1.14	0.05	0.25	0.00	-0.07	0.16
ED5L5D Health scale	91	-55	65	9.68	18.32	10	-3	20

Table E3. Neuropsychiatric scores as assessed by the GTI at baseline (V1) and after 12 months mepolizumab treatment (V2).

	Depression	
	V1 (%)	V2 (%)
None	34 (33.7)	66 (65.3)
Mild	15 (14.9)	9 (8.9)
Moderate	11 (10.9)	7 (6.9)
Severe	41 (40.6)	19 (18.8)
	Mood disturbance/mania	
	V1 (%)	V2 (%)
None	48 (47.5)	75 (74.3)
Mild	11 (10.9)	11 (10.9)
Moderate	14 (13.9)	8 (7.9)
Severe	28 (27.7)	7 (6.9)
	Insomnia	
	V1 (%)	V2 (%)
None	45 (44.6)	68 (67.3)
Without functional impairment	22 (21.8)	11 (10.9)
With functional impairment	34 (33.7)	22 (21.8)

Table E4: GTI 2.0: Changes in neuropsychiatric toxicity from V1 to V2.

N=101	V2 Depressive symptoms (%)				
V1 Depressive symptoms	Severe	Moderate	Mild	None	Total (%)
Severe	18 (43.9)	4 (9.8)	5 (12.2)	14 (34.1)	41 (40.6)
Moderate	1 (9.1)	1 (9.1)	0 (0)	9 (81.8)	11 (10.9)
Mild	0 (0)	0 (0)	2 (13.3)	13 (86.7)	15 (14.9)
None	0 (0)	2 (5.9)	2 (5.9)	30 (88.2)	34 (33.7)
Total (%)	19 (18.8)	7 (6.9)	9 (8.9)	66 (65.3)	101
	V2 Manic mood disturbance (%)				
V1 Manic mood disturbance	Severe	Moderate	Mild	None	Total (%)
Severe	5 (17.9)	6 (21.4)	2 (7.1)	15 (53.6)	28 (27.7)
Moderate	2 (14.3)	1 (7.1)	2 (14.3)	9 (64.3)	14(13.9)
Mild	0 (0)	0 (0)	2 (18.2)	9 (81.8)	11 (10.9)
None	0 (0)	1 (2.1)	5 (10.4)	42 (87.8)	48 (47.5)
Total (%)	7 (6.9)	8 (7.9)	11 (10.9)	75 (74.3)	101
	V2 Insomnia (%)				
V1 Insomnia	With functional impairment	Without functional impairment	None	Total (%)	
Insomnia with functional impairment	17 (50)	1 (2.9)	16 (47.1)	34 (33.7)	
Insomnia without functional impairment	2 (9.1)	6 (27.3)	14 (63.6)	22 (21.9)	
None	3 (6.7)	4 (8.0)	38 (84.4)	45 (44.6)	
Total (%)	22 (21.8)	11 (10.9)	68 (67.3)	101	

Table E5: Change in patient reported outcomes across the whole cohort from v1 to V2.

ACQ5 change:	
MCID met, improvement (≥0.5)	58 (59.2%)
Improvement but less than MCID (0 to 0.4)	23 (23.5%)
Worsening (≤0.1)	17 (17.3%)
Mini-AQLQ change:	
MCID met, improvement (≥-0.5)	58 (60.4%)
Improvement but less than MCID (0 to -0.49)	14 (14.6%)
Worsening (≤0.1)	24 (25.0%)
SGRQ change:	
MCID met, improvement (≥4)	68 (70.8%)
Improvement but less than MCID (0 to 3.9)	5 (5.2%)
Worsening (≤-0.1)	23 (24.0%)
MCID: Mini-AQLQ and ACQ5: MCID 0.5 , SGRQ MCID: 4 units,	

Table E6: Change in Hospital Anxiety and Depression scores across the whole cohort from v1 to V2.

HADs Anxiety score (n=97)	Number of patients in HADs category at V1	Number of patients at V2
0-7	40 (41.2%)	49 (50.5%)
8-10	20 (20.6%)	21 (21.6%)
>11	37 (38.1%)	27 (27.8%)
HADs Depression score (n=98)	Number of patients at V1	Number of patients at V2
0-7	55 (56.1%)	62 (63.9%)
8-10	15 (15.3%)	19 (19.6%)
>11	28 (28.6%)	17 (17.5%)
HADS: 0-7=normal, 8-10 mildly disturbed, >11 psychiatric impairment		

Figure E1. Glycaemic control on commencing mepolizumab from baseline (V1) and after 12 months mepolizumab (V2).

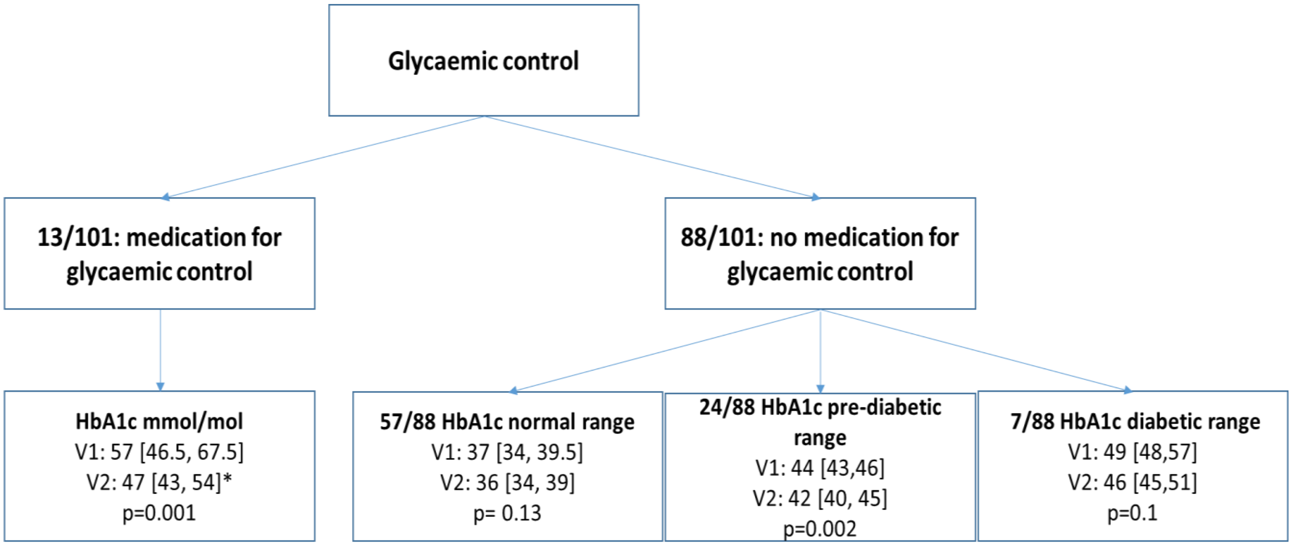

NICE criteria HbA1c: <41mmol/mol normal, 42-47 mmol/mol): increased risk pre-diabetic range, >48 mmol/mol): diabetic range (ref).
*At V2, 10 had no change in medication, 1 had a decrease and 2 an increase in medication for glycaemic control.

Figure E2: Correlation between toxicity reduction measured by AIS and change in (a) mini AQLQ from V1 to V2 (Spearman's correlation=-0.039 p=0.71), (b) SGRQ from V1 to V2 (Spearman's correlation=-0.18 p=0.088) and (c) ACQ 5 from V1 to V2 (Spearman's correlation=-0.009 p=0.93), (d) HADS depression score from V1 to V2 (Spearman's correlation=-0.17 p=0.10), (e) HADS anxiety score from V1 to V2 (Spearman's correlation =-0.18 p=0.086).

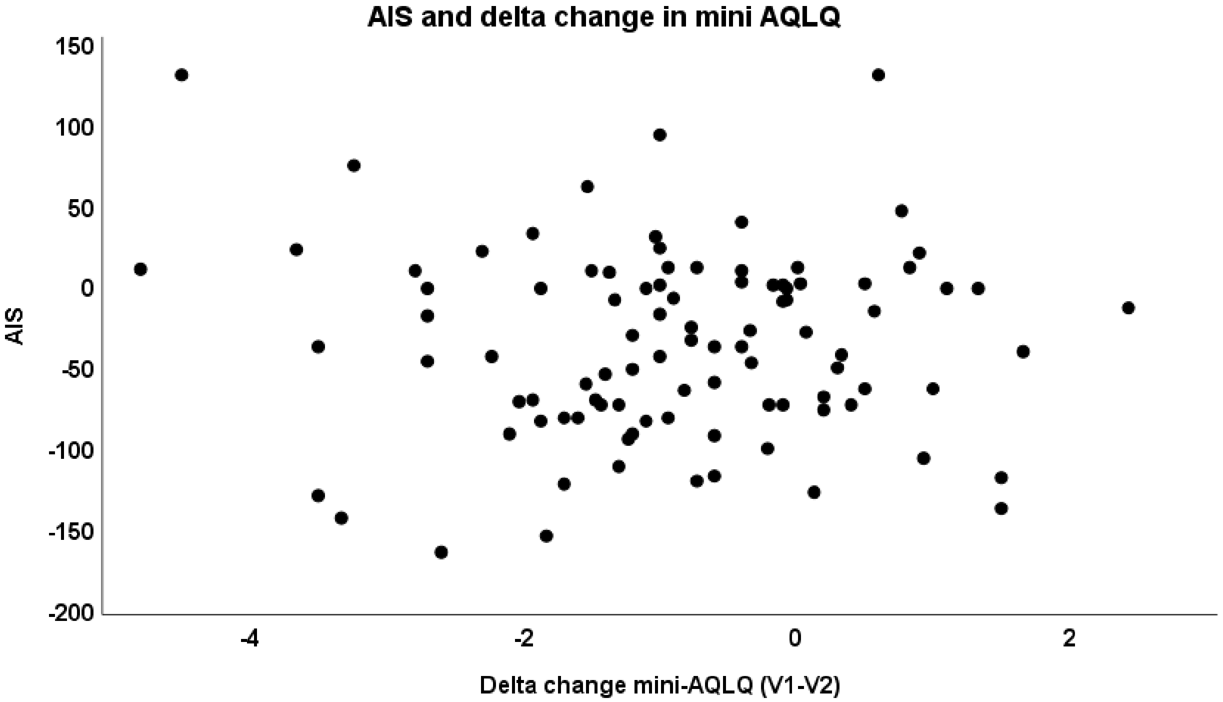

Figure E2 (a)

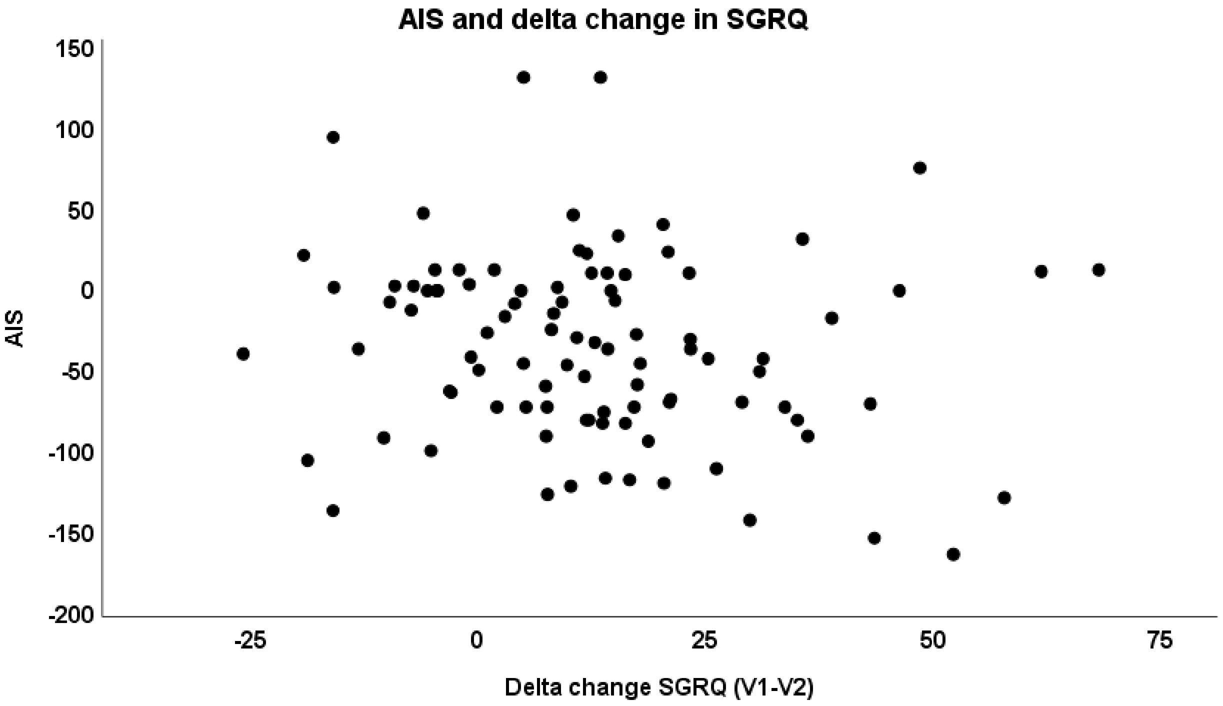

Figure E2 (b)

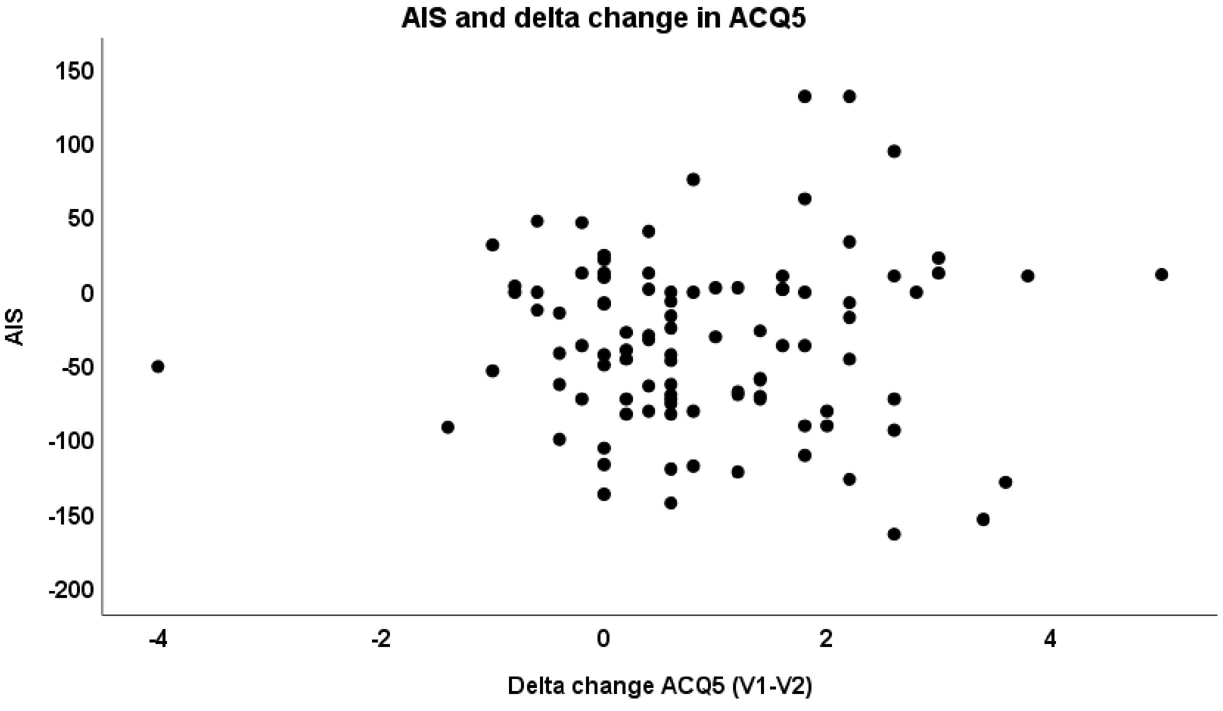

Figure E2 (c)

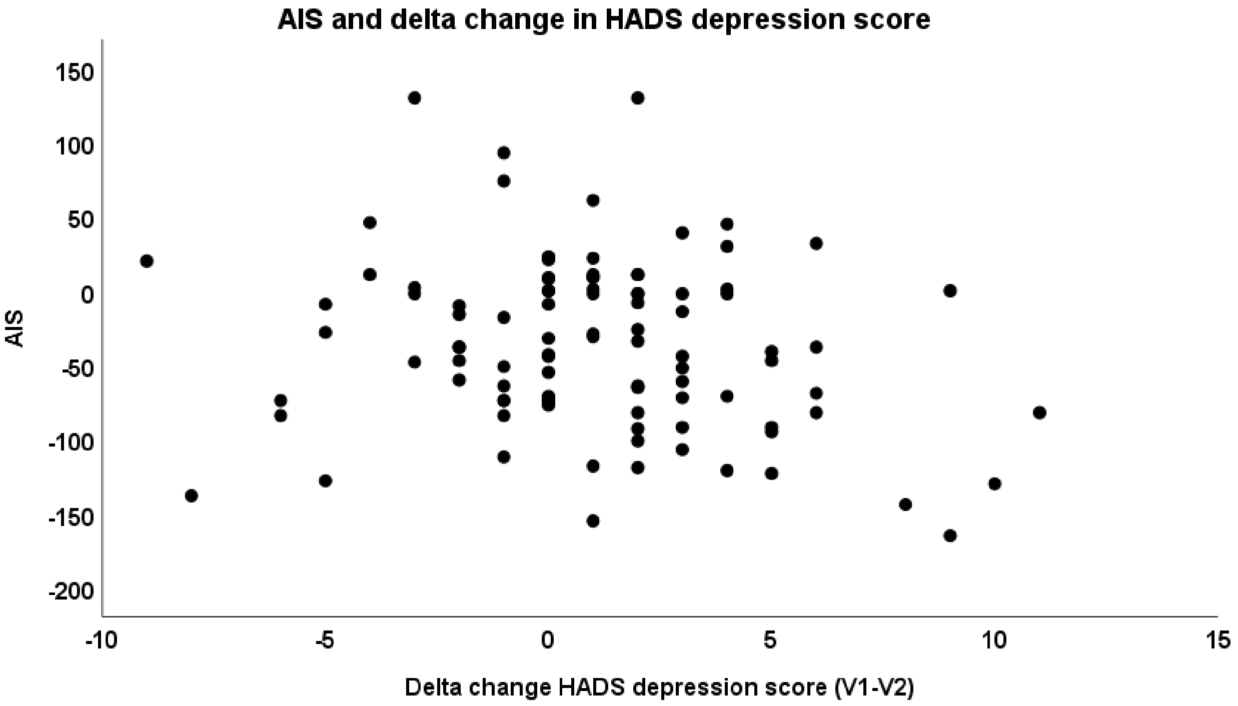

Figure E2 (d)

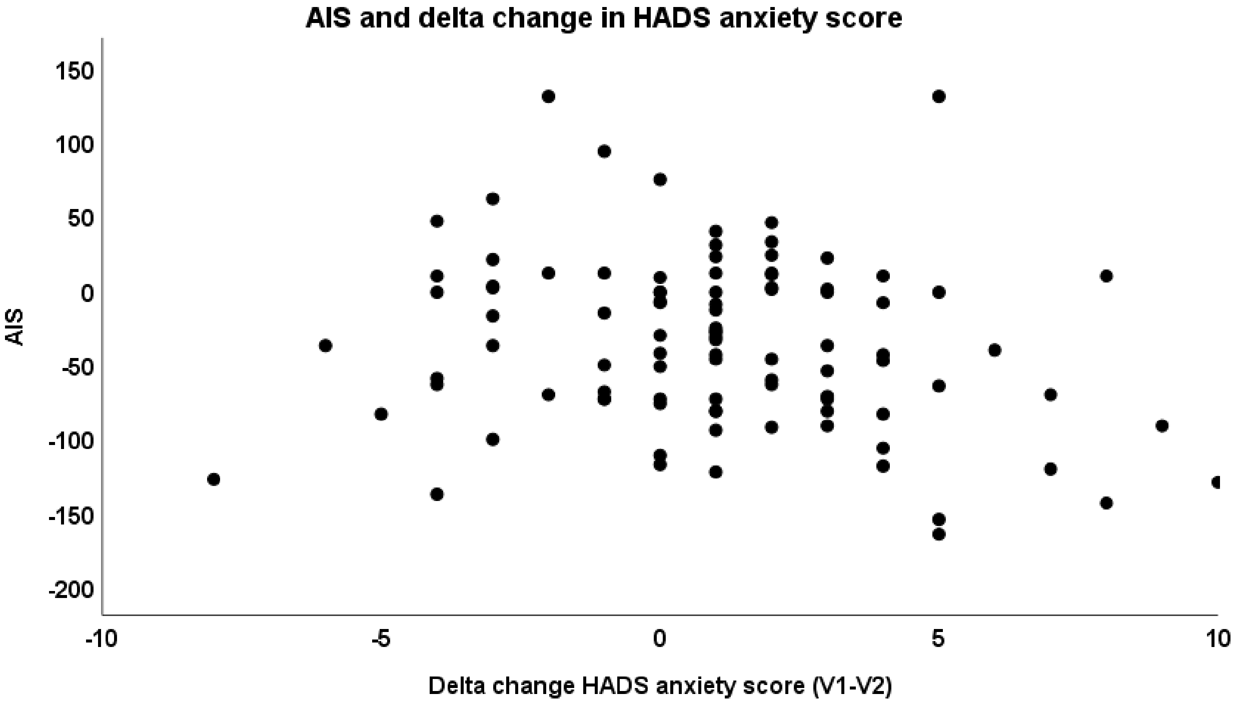

Figure E2 (e)

Figure E3: Correlation between toxicity reduction measured by AIS and (a) percentage decrease in prednisolone exposure from V1 to V2 (Spearman's correlation=-0.18, p=0.076), (b) and decrease in prednisolone exposure (mg) from V1 to V2 (Spearman's correlation=-0.17, p=0.093) and (c) decrease in prednisolone GC rescue courses from V1 to V2 (Spearman's correlation=-0.066, p=0.51).

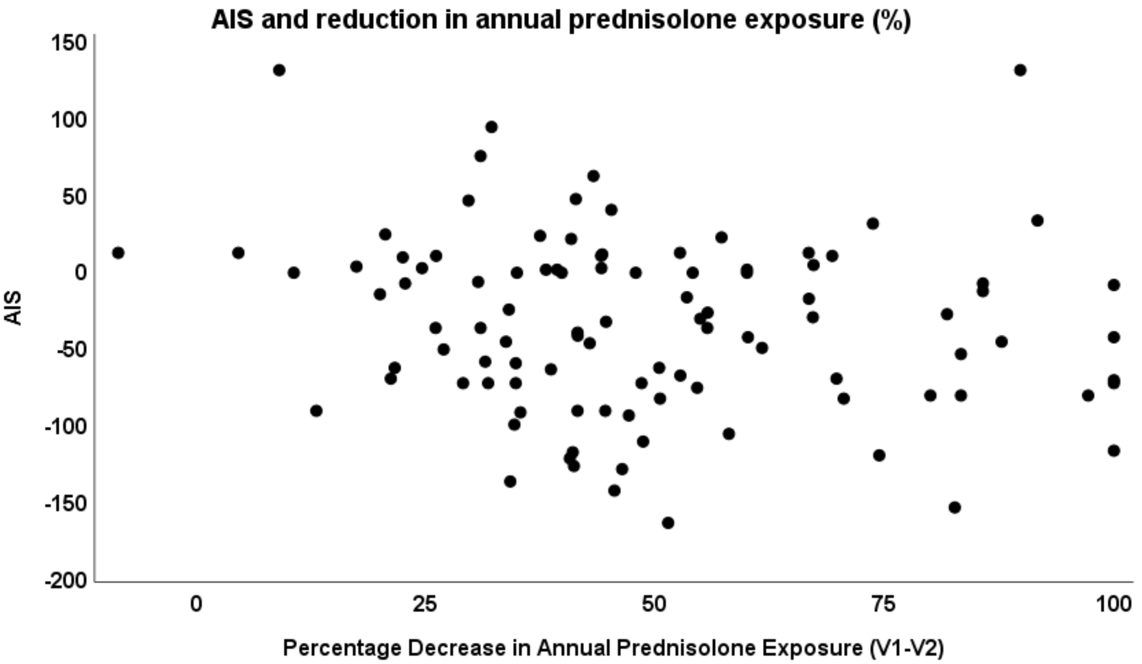

Figure E3 (a)

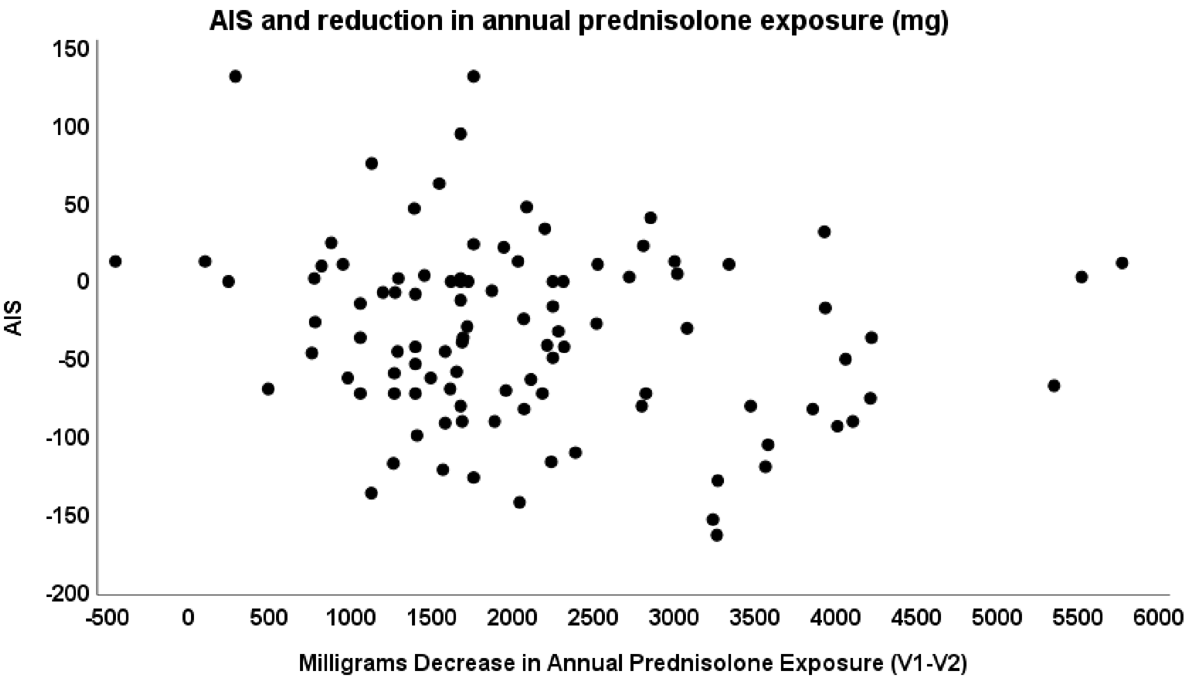

Figure E3 (b)

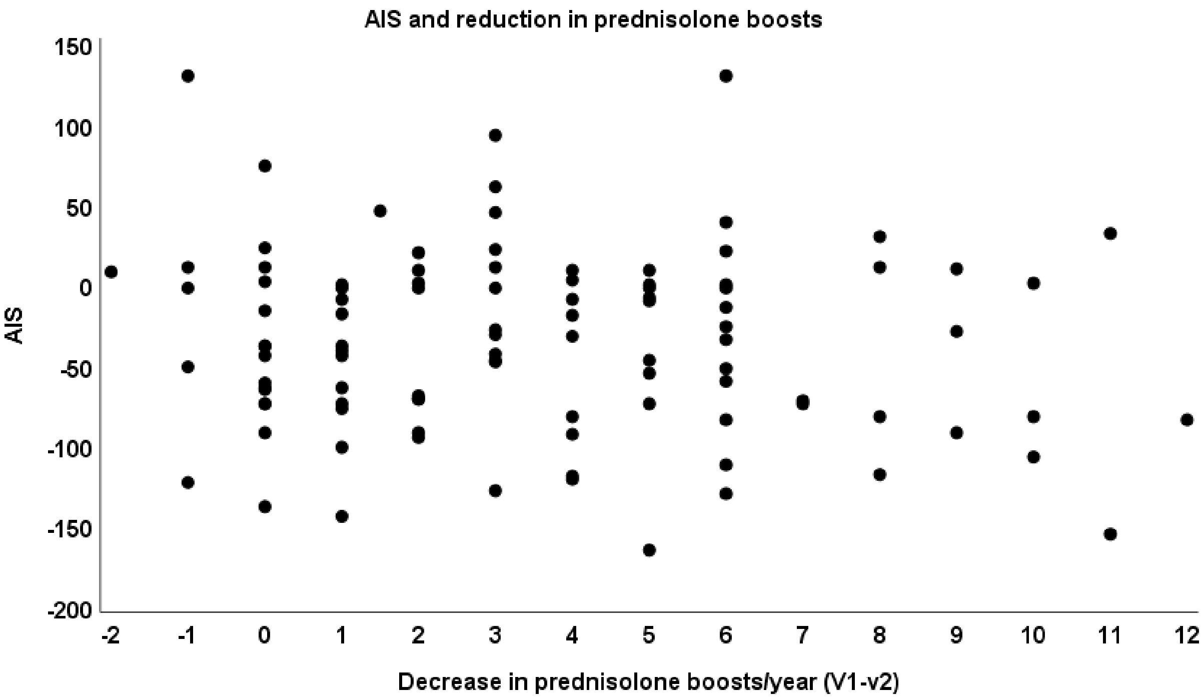
AIS and reduction in prednisolone boosts
150
100
50
0
-50
-100
-150
-200
AIS
-2
-1
0
1
2
3
4
5
6
7
8
9
10
11
12
Decrease in prednisolone boosts/year (V1-v2)

Figure E3 (c)

Figure E4: *Correlation between presence of new toxicities at V2 measured by CWS and (a) percentage decrease in prednisolone exposure from V1 to V2 (Spearman's correlation=0.02, p=0.84), (b) decrease in cumulative prednisolone exposure (mg) from V1 to V2 (Spearman's correlation=0.008, p=0.9) and (c) decrease in prednisolone* GC rescue courses *from V1 to V2 (Spearman's correlation=0.04, p=0.69).*

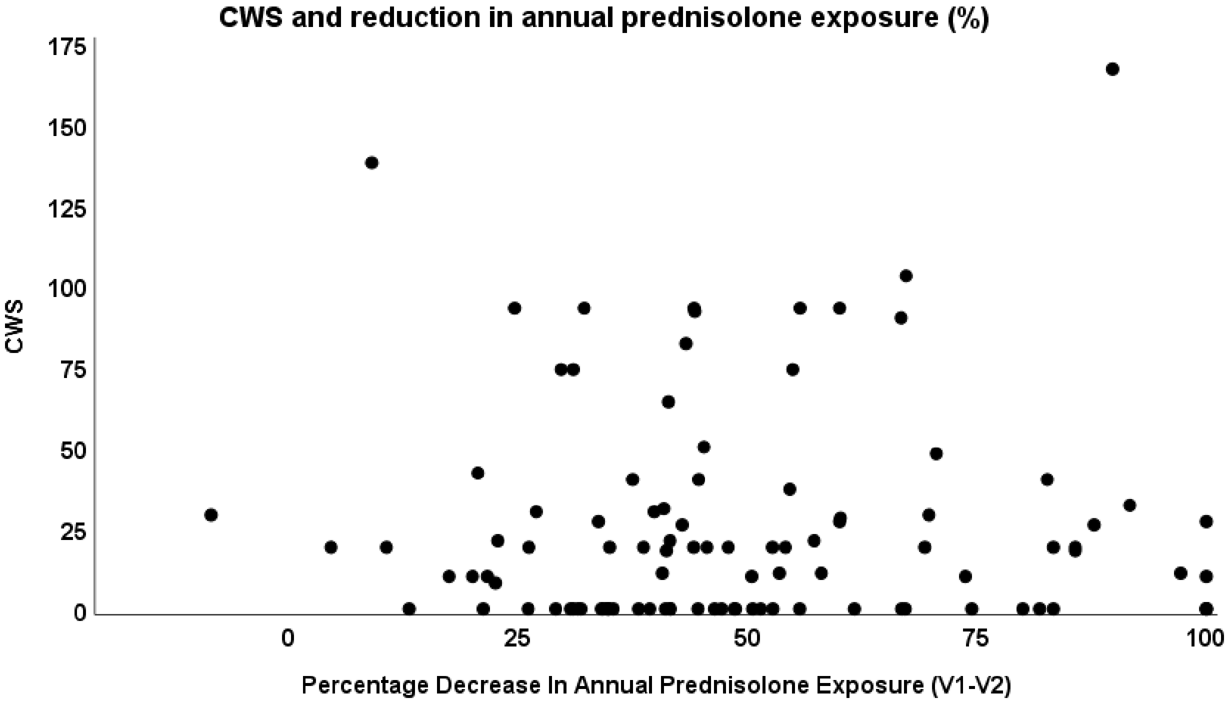

Figure E4 (a)

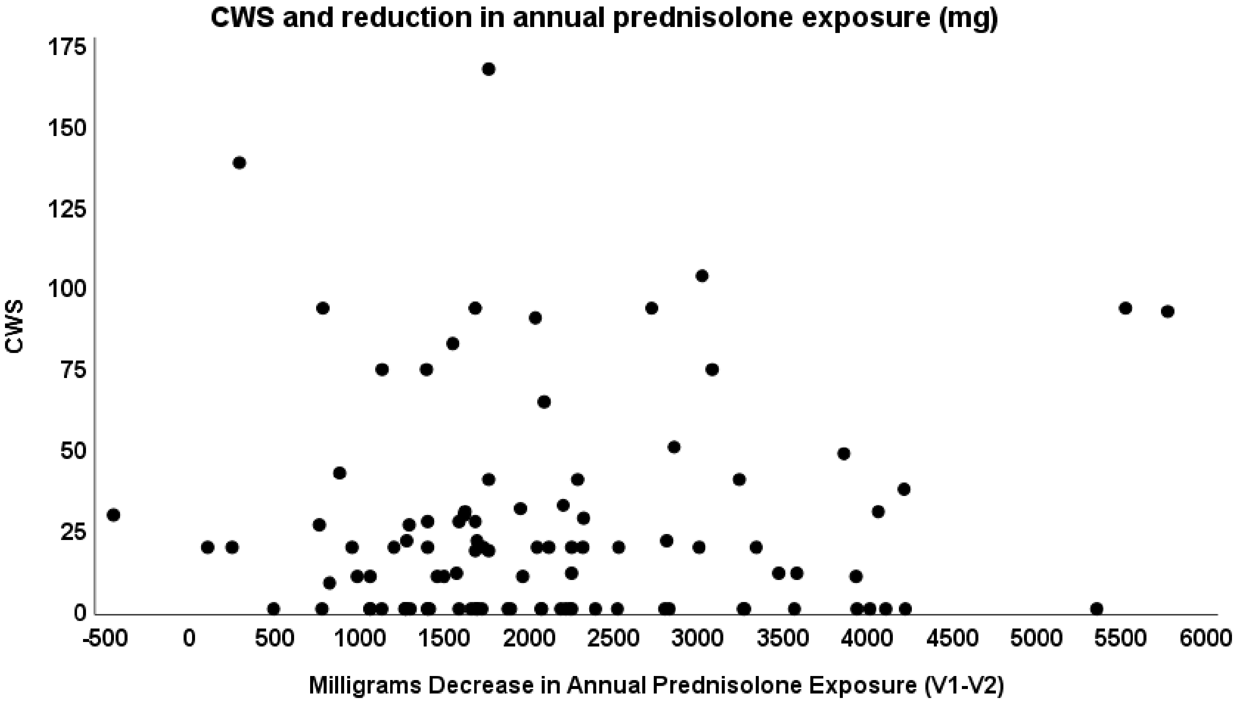

Figure E4 (b)

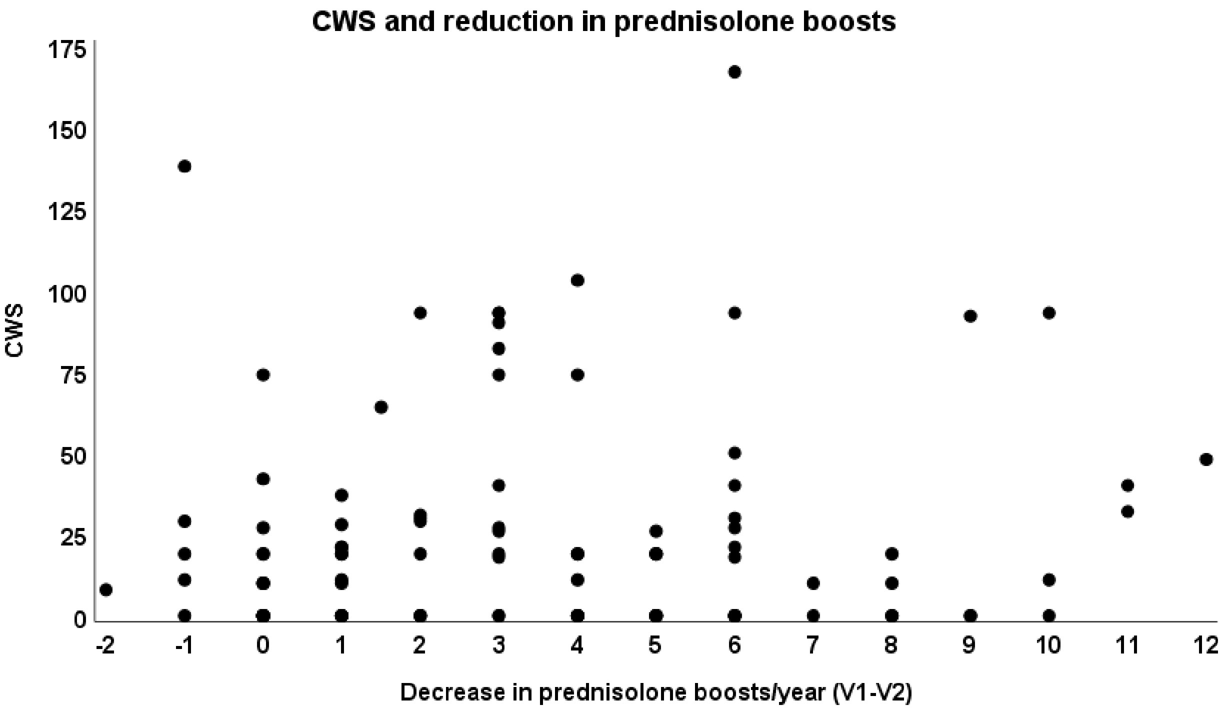
CWS and reduction in prednisolone boosts
CWS
175
150
125
100
75
50
25
0
-2
-1
0
1
2
3
4
5
6
7
8
9
10
11
12
Decrease in prednisolone boosts/year (V1-V2)

Figure E4 (c)

Figure E5. *Curve illustrating the percentage decrease in annual oral glucocorticoid dosing (X-axis) versus the probability of achieving the minimal clinically important difference (MCID) in the Aggregate Improvement Score of the Glucocorticoid Toxicity Index (Y-axis). The upper and lower bounds of the confidence intervals are shown by the shaded area. The curve, has wide confidence intervals, illustrating that percentage reduction of systemic glucocorticoid use is an inadequate indicator of glucocorticoid toxicity.*

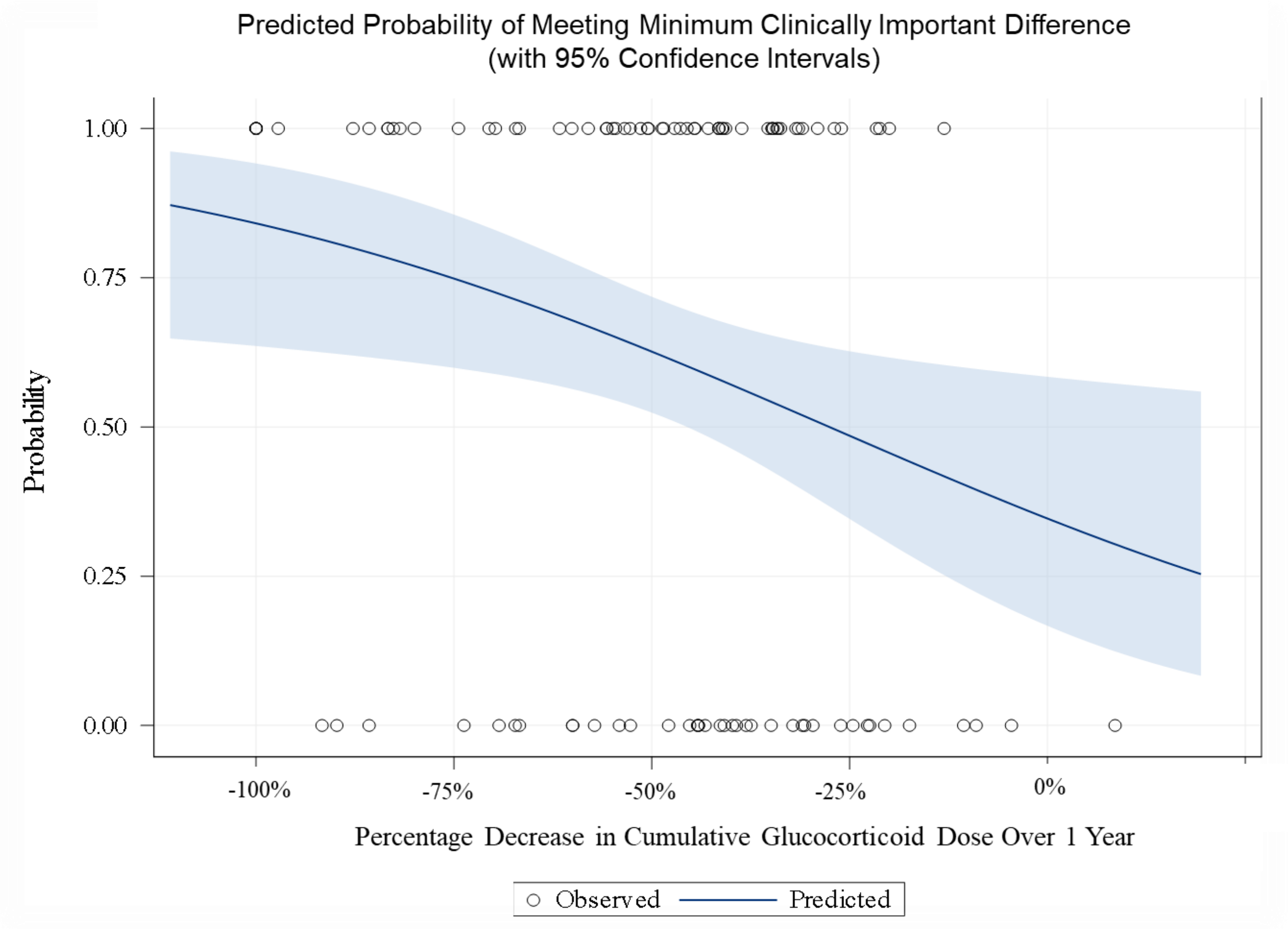

References:

E1. Miloslavsky EM, Naden RP, Bijlsma JWJ, Brogan PA, Brown ES, Brunetta P, et al. Development of a Glucocorticoid Toxicity Index (GTI) using multicriteria decision analysis. Ann Rheum Dis. 2016;76(3):1–4.

E2. Hansen P, Ombler F. A new method for scoring additive multi-attribute value models using pairwise rankings of alternatives. J Multi-Criteria Decis Anal [Internet]. 2008 May 1 [cited 2020 Mar 13];15(3–4):87–107. Available from: http://doi.wiley.com/10.1002/mcda.428

E3. McDowell PJ, Stone JH, Zhang Y, Honeyford K, Dunn L, Logan RJ, Butler CA, McGarvey LPA, Heaney LG. Quantification of Glucocorticoid-associated morbidity in Severe Asthma using the Glucocorticoid Toxicity Index. J Allergy Clin Immunol Pract. 2021;9(1):365-372

E4. EQ-5D-5L – EQ-5D. EQ-5D-5L. 2019. Available from: https://euroqol.org/eq-5d-instruments/eq-5d-5l-about/

E5. Juniper EF, Guyatt GH, Cox FM, Ferrie PJ, King DR. Development and validation of the Mini Asthma Quality of Life Questionnaire. Eur Respir J. 1999;14(1):32–8.

E6. Jones PW. St. George's respiratory questionnaire: MCID. In: COPD: Journal of Chronic Obstructive Pulmonary Disease. 2005. p. 75–9.

E7. Juniper EF, O'Byrne PM, Guyatt GH, Ferrie PJ, King DR. Development and validation of a questionnaire to measure asthma control. Eur Respir J. 1999;14(4):902–7.

E8. Juniper EF, Bousquet J, Abetz L, Bateman ED. Identifying "well-controlled" and "not well-controlled" asthma using the Asthma Control Questionnaire. Respir Med. 2006 Apr 1;100(4):616–21.

E9. Zigmond AS, Snaith RP. The Hospital Anxiety and Depression Scale. Acta Psychiatr Scand [Internet]. 1983 Jun [cited 2019 Nov 23];67(6):361–70. Available from: http://doi.wiley.com/10.1111/j.1600-0447.1983.tb09716.x

E10. Heaney LG, Conway E, Kelly C, Gamble J. Prevalence of psychiatric morbidity in a difficult asthma population: Relationship to asthma outcome. Respir Med. 2005 Sep 1;99(9):1152–9.
