# Supplementary material for: Glucocorticoid toxicity reduction with mepolizumab using the Glucocorticoid Toxicity Index
Source: Eur Respir J. 2022 Jan 20;59(1):2100160. doi: 10.1183/13993003.00160-2021 (PMC8770919; doi:10.1183/13993003.00160-2021)
Supplement: Supplementary file 2 [file ERJ-00160-2021.Shareable.pdf]

SHAREABLE PDF

Glucocorticoid toxicity reduction with mepolizumab using the Glucocorticoid Toxicity Index

P. Jane McDowell [1], John H. Stone [2], Yuqing Zhang [2], Kirsty Honeyford [3], Louise Dunn [3], R. Jayne Logan [3], Lorcan P.A. McGarvey [1], Claire A. Butler [3] and Liam G. Heaney [1]

[1]Wellcome-Wolfson Centre for Experimental Medicine, School of Medicine, Dentistry, and Biological Sciences, Queen's University Belfast, Belfast, UK. [2]Division of Rheumatology, Allergy, and Clinical Immunology, Massachusetts General Hospital, Harvard Medical School, Boston, MA, USA. [3]Dept of Respiratory Medicine, Belfast City Hospital, Belfast Health and Social Care Trust, Belfast, UK.

Corresponding author: Liam Heaney (l.heaney@qub.ac.uk)

Check for updates

Shareable abstract (@ERSpublications)
Anti-T2 biologics decrease glucocorticoid requirements in severe eosinophilic asthma, but glucocorticoid reduction does not correlate with glucocorticoid toxicity reduction in individuals. Assessment of glucocorticoid toxicity reduction is crucial when considering response to biologics. https://bit.ly/3goyIRd

Cite this article as: McDowell PJ, Stone JH, Zhang Y, *et al*. Glucocorticoid toxicity reduction with mepolizumab using the Glucocorticoid Toxicity Index. *Eur Respir J* 2022; 59: 2100160 [DOI: 10.1183/13993003.00160-2021].

This single-page version can be shared freely online.

Copyright ©The authors 2022.

This version is distributed under the terms of the Creative Commons Attribution Licence 4.0.

This article has supplementary material available from erj.ersjournals.com

Received: 18 Jan 2021
Accepted: 2 June 2021

Abstract

Background Reduction in glucocorticoid exposure is the primary benefit of new biologic treatments in severe asthma, but there is currently no evidence that reduction in glucocorticoid exposure corresponds to a proportionate reduction in associated toxicity.

Objectives To use the validated Glucocorticoid Toxicity Index (GTI) to assess change in glucocorticoid toxicity after 12 months treatment with mepolizumab, and compare toxicity change to glucocorticoid reduction and change in patient-reported outcome measures (PROMs).

Methods A longitudinal, real-world prospective cohort of 101 consecutive patients with severe asthma commenced on mepolizumab in a specialist UK regional severe asthma clinic. GTI toxicity assessment, cumulative glucocorticoid exposure and PROMs were recorded on commencing mepolizumab (V1), and after 12 months treatment (V2).

Results There was significant reduction in oral glucocorticoid exposure (V1 median 4280 mg prednisolone per year (interquartile range 3083–5475 mg) *versus* V2 2450 mg prednisolone per year (1243–3360 mg), p<0.001). Substantial improvements in individual toxicities were observed, but did not correlate with oral glucocorticoid reduction. Mean±SD GTI aggregate improvement score (AIS) was −35.7±57.8 with a wide range in toxicity change at individual patient level (AIS range −165 to +130); 70% (71 out of 101) had a reduction in toxicity (AIS <0); 3% (three out of 101) had no change (AIS=0); and 27% (27 out of 101) an increase in overall toxicity. 62% (62 out of 101) of patients met the AIS minimally clinically important difference of ⩽−10, but AIS did not correlate with glucocorticoid reduction or change in PROMs.

Conclusion Mepolizumab resulted in substantial oral glucocorticoid reduction, but this did not correlate with reduction in oral glucocorticoid toxicity, which varies widely at the individual patient level. Oral glucocorticoid reduction is not a comprehensive measure of response to mepolizumab.
